# Supplementary material for: IRSp53 controls plasma membrane shape and polarized transport at the nascent lumen in epithelial tubules
Source: Nat Commun. 2020 Jul 14;11:3516. doi: 10.1038/s41467-020-17091-x (PMC7360740; doi:10.1038/s41467-020-17091-x)
Supplement: Supplementary file 1 — Supplementary Information [file 41467_2020_17091_MOESM1_ESM.pdf]

IRSp53 controls plasma membrane shape and polarized transport at the nascent lumen in epithelial tubules

Bisi S., et al

SUPPLEMENTARY INFORMATION

- Supplementary Tables 1-2

- Supplementary Figures

Supplementary Table 1

**Primary antibodies**	Application/ Dilution
IRSp53, mouse (IFOM)	WB 1:150; IF 1:150; IHC 1:150/1:200
IRSp53, rabbit (SIGMA, HPA023310)	IHC 1:50
IRSp53, rabbit(Hans-Jürgen Kreienkamp Lab)	WB 1:200; IF 1:100; IHC 1:30
Vinculin, mouse (SIGMA, V9131)	WB 1:1000
GFP, rabbit (SIGMA, G1544)	WB 1:500
GFP, mouse (Abcam, ab6556)	Immuno-EM 1:100
GFP, chicken (Abcam, ab13970)	IF 1:1000
anti-GP135/PODXL, mouse (DSHB, Iowa University, 3F2/D8)	WB 1:500; IF 1:400
Eps8, mouse (BD-Transduction Lab, 610144)	WB 1:1000; IF 1:1000 (Caco2 cysts 1:100)
anti-RAB7, rabbit (Cell Signaling, 2094)	IF 1:150
ZO1, rabbit (Invitrogen, 402200)	IF 1:400 (Caco2 cysts 1:100)
ZO-1, rabbit (Genetex, GTX108592)	IHC 1:100
PKC ζ, rabbit (SCBT, sc-216)	IF (Caco2 cyts) 1:100; IHC 1:100
ß-catenin, rabbit (SIGMA, PLA2030)	IF 1:500 (Caco2 cysts 1:100); Immuno-EM 1:100
anti-Myc 9E10, mouse (IFOM)	WB 1:1000; IF 1:1000
EEA1, goat (SCBT, sc-6415)	IF 1:150
LAMP1, rabbit (SIGMA, L1418)	IF 1:100
Giantin, rabbit (BioLegend, 924302)	IF 1:100
Flag, mouse (SIGMA, F3165)	WB 1:1000; IF 1:1000; IP 5µg/mg
Rab35, rabbit (Cell Signaling, 9690)	WB 1:500
Tubulin, mouse (SIGMA, T5168)	WB 1:1000; IF 1:1000
Acetyl-α-tubulin, mouse (Abcam, Ab24610)	IF 1:500
Endomucin, rat (Abcam, ab106100)	IHC 1:100

Supplementary Table 2

**Secondary antibody**	Application/ Dilution
anti-rabbit IgG, HRP-linked antibody (Cell Signaling, 7074)	WB 1:2500
anti-mouse IgG, HRP-linked antibody (Cell Signaling, 7076)	WB 1:2500
anti-rabbit Fab' fragments coupled to 1.4nm gold particles, goat (Nanoprobes, #2004)	Immuno-EM 1:100
anti-mouse Fab' fragments coupled to 1.4nm gold particles, goat (Nanoprobes, #2002)	Immuno-EM 1:100
Alexa Fluor 488 Donkey anti rabbit (ThermoFisher, A-21206)	IF 1:400 (Caco2 cysts 1:100)
Alexa Fluor 488 Donkey anti mouse (ThermoFisher, A-21202)	IF 1:400 (Caco2 cysts 1:100)
Alexa Fluor 488 Goat anti chicken (Thermofisher, A-11039)	IF 1:400
Alexa Fluor 647 Donkey anti rabbit (ThermoFisher, A-31573)	IF 1:400 (Caco2 cysts 1:100)
Alexa Fluor 647 Donkey anti mouse (ThermoFisher, A-31571)	IF 1:400 (Caco2 cysts 1:100)
Cy3 Donkey anti rabbit (Jackson Immunoresearch, 711-165-152)	IF 1:400 (Caco2 cysts 1:100)
Cy3 Donkey anti mouse (Jackson Immunoresearch, 715-165-150)	IF 1:400 (Caco2 cysts 1:100)
Cy3 Donkey anti goat (Jackson Immunoresearch, 705-165-147)	IF 1:400
Phalloidin TRITC (SIGMA, P1951)	IF 1:50 (Caco2 cysts 1:10)
Phalloidin FITC (SIGMA, P5282)	IF 1:50 (Caco2 cysts 1:10)

Supplementary Figure 1

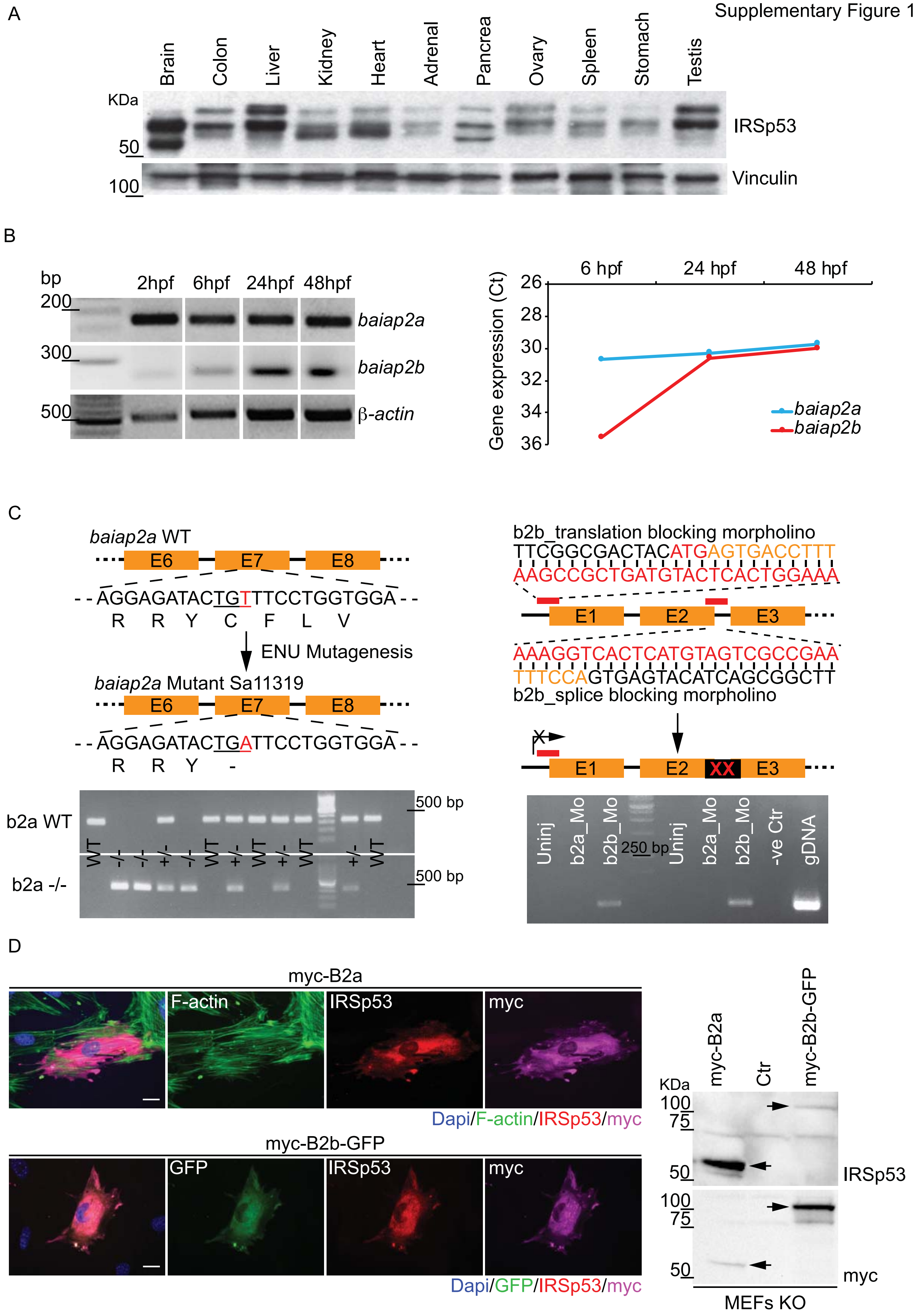

Supplementary Figure 1

Expression of IRSp53 in murine tissues and of orthologues Baiap2A and 2B in zebrafish

A) IRSp53 is expressed in different mouse organs. Lysates derived from the indicated organs were processed by immunoblotting to analyse the expression levels of IRSp53 and vinculin.

B) *baiap2a* and *baiap2b* expression analysis during zebrafish embryogenesis. Total RNA was extracted from embryos at different developmental stages and retro-transcribed. The same amount of cDNA (500 ng) was used as template for Reverse Transcriptase-PCR (RT-PCR). Left: Semi-quantitative RT-PCR. Right: qRT-PCR analysis, data are expressed as Ct after normalization with the house keeping gene (β-actin).

C) Schematic of the genomic regions of the zebrafish genes *baiap2a* (on the left) and *baiap2b* (on the right) targeted by ENU mutagenesis or morpholinos. The mutation in *baiap2a* generates a premature stop codon in exon 7. The *baiap2b* translation-blocking morpholino recognizes a region in the 5'-UTR that causes a translation block; the *baiap2b* splice-blocking morpholino was designed on the junction between exon 2 and intron 2 (E2I2) of *baiap2b* and causes the retention of an intronic fragment in the mature mRNA. Below, the gel electrophoresis related to the genotyping of *baiap2a* mutant embryos (left) and to the specificity of the splice-blocking morpholino-*baiap2b* (right).

D) The rabbit polyclonal anti-IRSp53 antibody recognises the gene products of *baiap2a* and *baiap2b*. Left: Mouse embryonic fibroblasts derived from IRSp53-KO mice (MEFs KO) were transfected with either myc-Baiap2a (upper panels) or myc-Baiap2b-GFP (lower panels). Cells were fixed and stained as indicated. Scale bar, 25 μm. Right: Lysates from control MEFs KO (Ctr) and MEFs KO transiently expressing either myc-Baiap2a or myc-Baiap2b-GFP were immunoblotted with the indicated antibodies. Arrows indicate myc-Baiap2a and myc-Baiap2b-GFP respectively.

Supplementary Figure 2

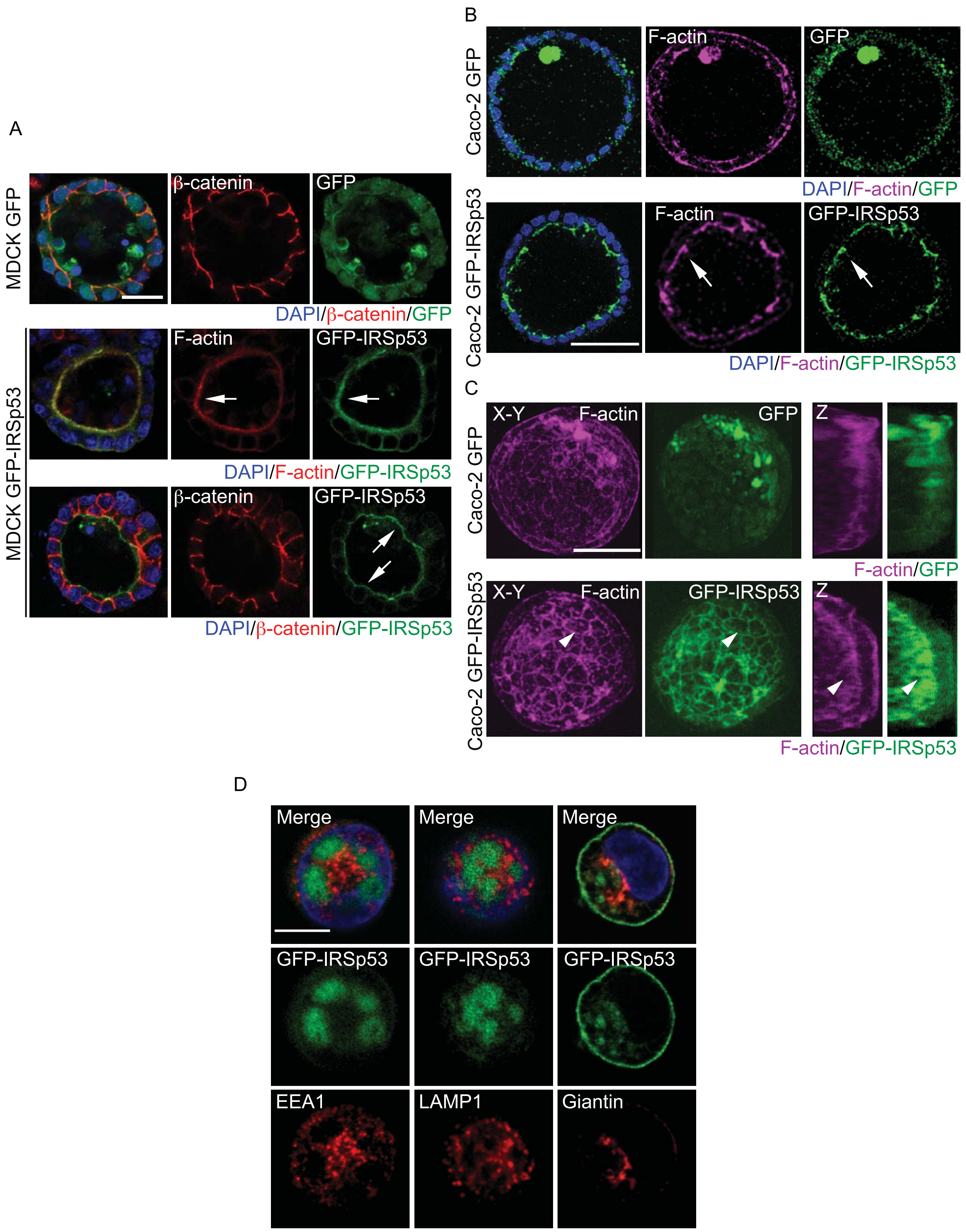

Supplementary Figure 2

IRSp53 localization in 2D epithelial cells and 3D cysts

A) MDCK-expressing GFP or GFP-IRSp53 were seeded as single cells on Matrigel and left growing to form 3D cysts. Cysts were fixed and processed for epifluorescence to visualize GFP or GFP-IRSp53 (green) and stained with rhodamine phalloidin (red) to visualized F-actin or anti-β-catenin (red) and Dapi (blue). Arrows indicate IRSp53 enrichment on the luminal side of the cyst. Scale bar, 18 μm.

B) Caco-2 cells, expressing either GFP or GFP-IRSp53, were embedded as single cells into Matrigel/Collagen matrix and left growing to form 3D cysts. Cysts were fixed and processed for epifluorescence to visualize GFP or GFP-IRSp53 (green), and stained with SiR-actin to detect F-actin (magenta) and Dapi (blue). Arrows indicate IRSp53 and F-actin enrichment at the luminal side of the cyst. Scale bar, 70 μm.

C) Maximum projection (X-Y plane, left panels) and 3D reconstruction (Z plane, right panels) of confocal Z-stack acquisitions of the cysts shown in D). Arrowheads indicate IRSp53 and F-actin enrichment at cell-cell junction (X-Y plane) and at the luminal side of the cyst (Z plane). Scale bar, 70 μm.

D) MDCK expressing GFP-IRSp53 were seeded as single cells on Matrigel and fixed after 5h. Cysts were processed for epifluorescence to visualize GFP-IRSp53 (green) and stained with either anti-EEA1 (red), anti-LAMP1 (red) or anti-Giantin (red) and Dapi (blue). Scale bar, 10 μm.

Supplementary Figure 3

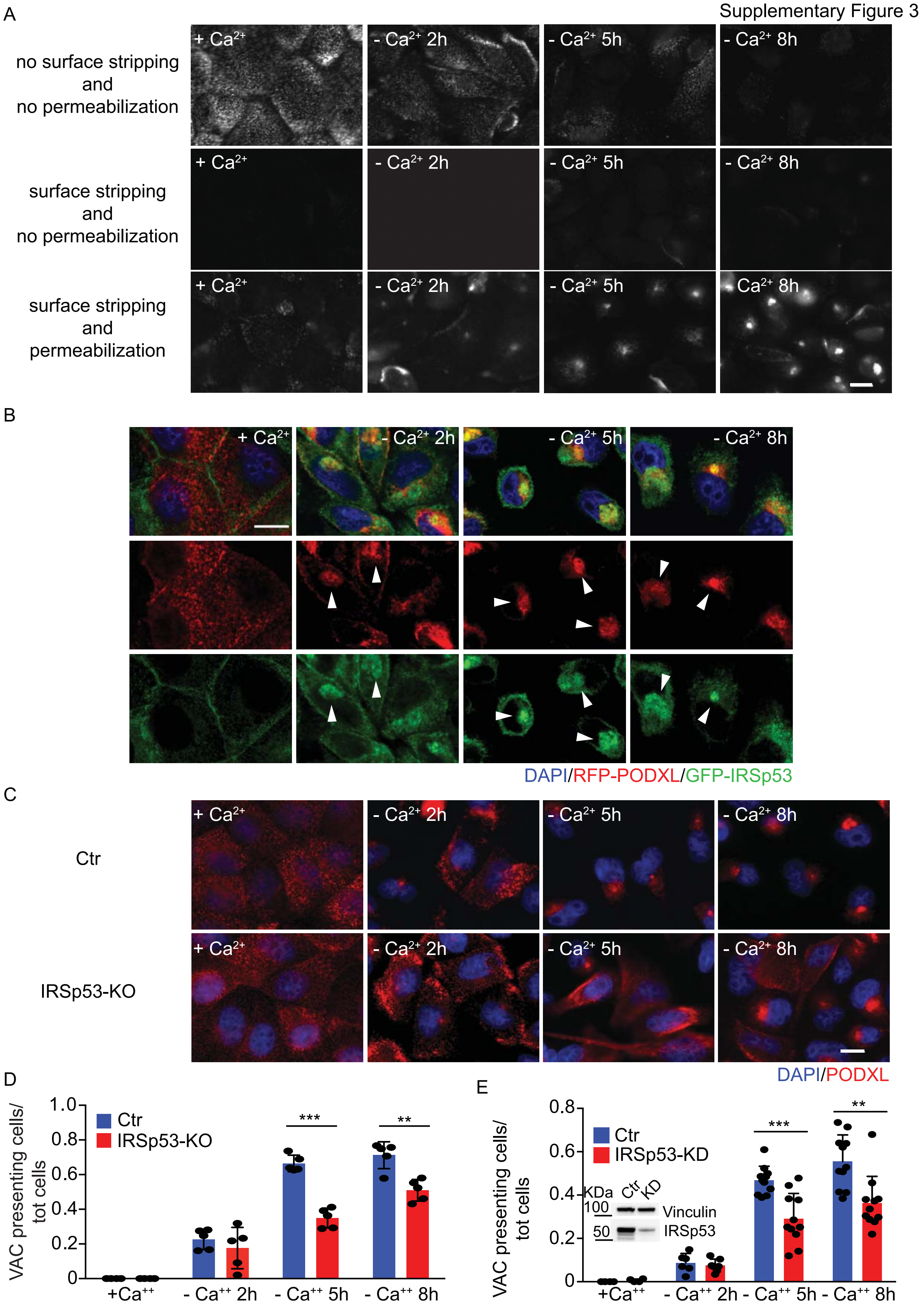

Supplementary Figure 3

IRSp53 impacts on Podocalyxin trafficking to the apical compartment

A) PODXL re-localization assays at VAC (Vacuolar Apical Compartment)[1,2]. MDCK monolayers, pre-incubated with anti-PODXL antibody for 1h, were grown without Ca^{2+} for the indicated time points and fixed without surface stripping (acidic-based removal of cell surface bound proteins) and permeabilization, or subjected to surface stripping (0.5% acetic acid pH3 + 0.5 M NaCl) to wash out residual PODXL antibody bound to the cell surface. Cells were then fixed and stained with anti-PODXL antibody either without (surface stripping and no permeabilization) or with (surface stripping and permeabilization) previous permeabilization (PBS 1X, 0,1% Triton X-100, 10min), as indicated. Scale bar, 10 μm.

B) IRSp53 and PODXL colocalize at VAC. GFP-IRSp53 and RFP-PODXL stably expressing MDCK monolayers were grown without Ca^{2+} for the indicated time points and processed for epifluorescence to visualize GFP-IRSp53 (green) and RFP-PODXL (red) or stained with DAPI (blue) after fixation. Arrowheads indicate colocalization of GFP-IRSp53 and RFP-PODXL at VAC. Scale bar, 10 μm.

C) IRSp53 depletion delays PODXL re-localization at VAC. MDCK control (Control) or IRSp53-KO monolayers were grown without Ca^{2+} for the indicated time points and permeabilized after fixation. Cells were stained with anti-PODXL antibody (red) and DAPI (blue). Scale bar, 10 μm.

D) Quantification of PODXL re-localization at VAC, expressed as VAC-presenting cells/ total number of cells. Data are expressed as mean ± SD. At least 100 cells/time point/four different fields were analysed in n= 1 experiment. P value, Student's t-test two-tailed. $^{**}p < 0.01$; $^{***}p<0.001$. Source data are provided as a Source Data file.

E) Quantification of PODXL re-localization at VAC in MDCK Ctr vs IRSp53-KD monolayer (not shown) expressed as VAC presenting cells/ total number of cells. Data are expressed as mean ± SD. At least 100 cells/time point/four different fields were analysed in n= 2 independent experiments. P value, Student's t-test two-tailed. $^{**}p < 0.01$; $^{***}p<0.001$. Source data are provided as a Source Data

file. Inset: the level of expression of IRSp53 and vinculin were analysed by immunoblotting to verify IRSp53 downregulation.

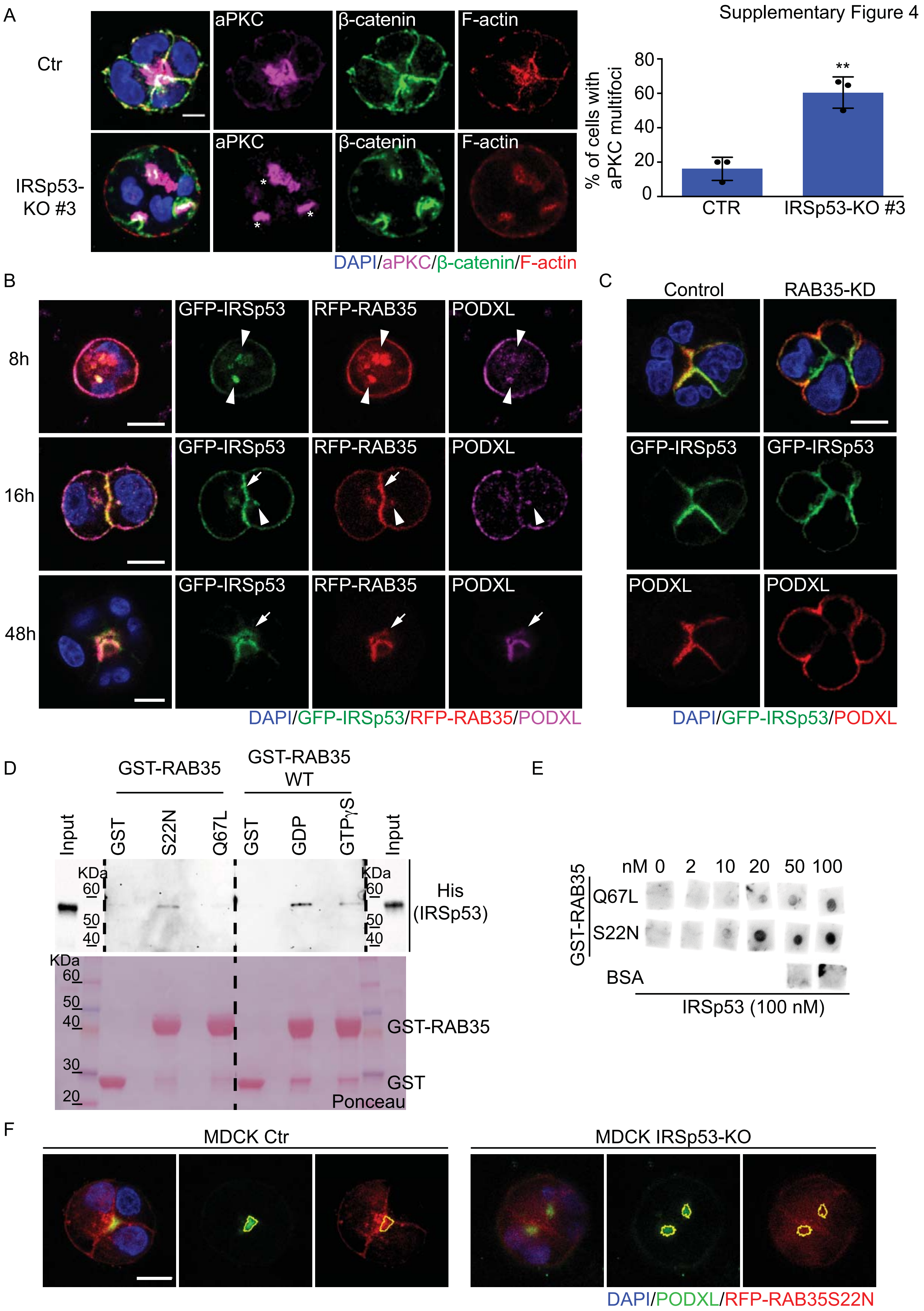

Supplementary Figure 4
A
Ctr
IRSp53-KO #3
aPKC
β-catenin
F-actin
DAPI/aPKC/β-catenin/F-actin
% of cells with aPKC multifoci
80
60
40
20
0
**
CTR
IRSp53-KO #3
B
8h
16h
48h
GFP-IRSp53
RFP-RAB35
PODXL
DAPI/GFP-IRSp53/RFP-RAB35/PODXL
C
Control
RAB35-KD
GFP-IRSp53
PODXL
DAPI/GFP-IRSp53/PODXL
D
GST-RAB35
GST-RAB35 WT
Input
GST
S22N
Q67L
GST
GDP
GTPγS
Input
KDa
60
50
40
His (IRSp53)
30
20
GST-RAB35
GST
Ponceau
E
nM 0 2 10 20 50 100
GST-RAB35
Q67L
S22N
BSA
IRSp53 (100 nM)
F
MDCK Ctr
MDCK IRSp53-KO
DAPI/PODXL/RFP-RAB35S22N

Supplementary Figure 4

IRSp53 controls aPKC distribution, colocalizes and interacts directly with RAB35

A) Caco-2 control (Ctr) or IRSp53-KO (#3), were embedded as single cells in Matrigel/Collagen matrix and left growing for 48/72h. Cysts were fixed and stained with anti aPKC (magenta), anti-β-catenin (green), rhodamine-phalloidin to detect F-actin (red) and Dapi (blue). Asterisks indicate aPKC multi-foci in IRSp53-KO cyst. Scale bar, 10 μm. Right: Quantification of cysts with multiple aPKC foci. Data are expressed as mean ± SD. Four-cells stage cysts were analysed. At least 15 cysts/experiment were analysed in n= 3 independent experiments. P value, Student's t-test two-tailed. **p < 0.01. Source data are provided as a Source Data file.

B) Time course of IRSp53, RAB35, and PODXL localization in the early phases of cystogenesis. Cysts from MDCK expressing GFP-IRSp53 and RFP-RAB35 cells were fixed at the indicated time points, processed for epifluorescence to visualize GFP-IRSp53 (green) and RFP-RAB35 (red), and stained with anti-PODXL (magenta) and Dapi (blue). Arrowheads indicate IRSp53, RAB35, and PODXL colocalization in vesicle-like structures. Arrows indicate IRSp53 and RAB35 preceding PODXL re-localization on AMIS and the enrichment of the three proteins on the luminal side at later time points. Scale bar, 10 μm.

C) RAB35 silencing does not alter IRSp53 localization during cystogenesis. GFP-IRSp53 expressing MDCK cells treated with a scramble siRNA (Ctr) or a siRNA against RAB35 (RAB35-KD) were seeded as single cells on Matrigel and fixed after 24 hours. Cysts were processed to visualize GFP-IRSp53 (green), stained with anti-PODXL (red) and Dapi (blue). Scale bar, 10 μm.

D) Recombinant purified His-IRSp53 (1 μM) was incubated in IVB buffer (see Methods section) with 2.5 μM of recombinant purified GST-RAB35S22N or GST-RAB35Q67L or GST-RAB35WT previously loaded with GDP or GSTγS (see Methods). Inputs and IVBs were loaded on SDS PAGE, analysed by Ponceau staining to detect GST or GST-RAB35 purified proteins, and subjected to WB analysis with the indicated antibody.

E) Recombinant purified GST-RAB35Q67L and GST-RAB35S22N proteins were spotted at the indicated concentrations on nitrocellulose filters. BSA was used as negative control. Nitrocellulose membranes were incubated with recombinant purified IRSp53 protein (100 nM) and immunoblotted with an anti-IRSp53 specific antibody.

F) Control (WT) and IRSp53 KO MDCK expressing mRFP-RAB35-S22N were seeded on top of Matrigel to generate cysts. Early stage cysts were fixed, stained with anti-podocalyxin (PODXL) to detect the AMIS and processed for epifluorescence to detect mRFP-RAB35-S22N. The images are representative of 21 cysts that were analysed in 2 independent experiments. Scale bar, 10 µm.

Supplementary Figure 5

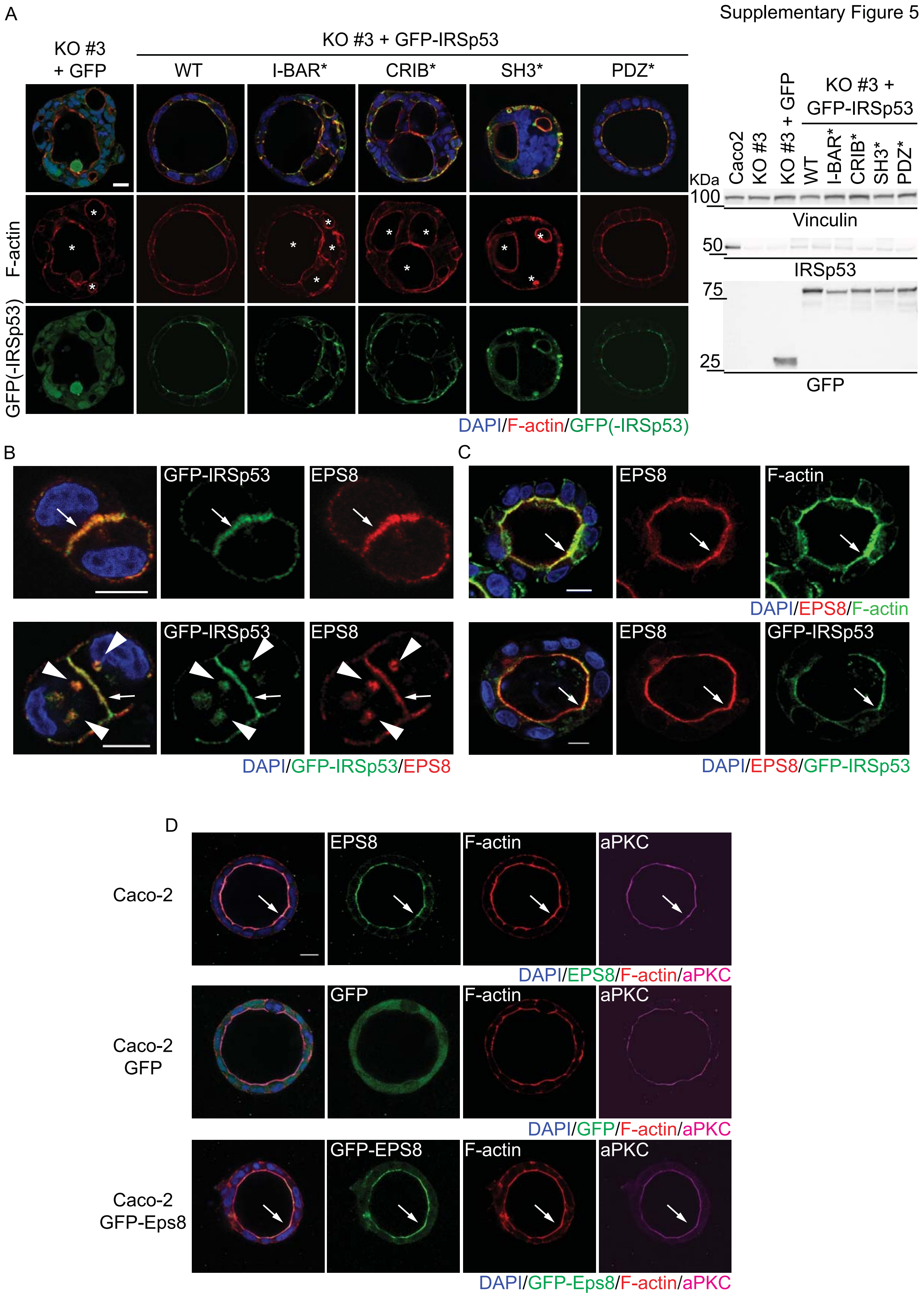

Supplementary Figure 5

Structure-Function analysis of IRSp53 and Eps8 localization in epithelial cysts

A) Caco-2 IRSp53-KO #3 stably-infected with GFP or murine GFP-IRSp53 WT, or I-BAR*, CRIB*, SH3*, PDZ* mutants were embedded as single cells in Matrigel/Collagen matrix and fixed after 7 days. Cysts were processed for epifluorescence to visualize GFP or GFP-IRSp53 (green) and stained with rhodamine-phalloidin to detect F-actin (red) and Dapi (blue). Asterisks indicate multiple lumens. Scale bar, 20 µm. Right: The expression levels of IRSp53, GFP, GFP-IRSp53 WT and mutants, and vinculin were analysed by immunoblotting to verify the depletion of endogenous IRSp53 and the expression of the GFP-fusion constructs.

B) MDCK infected with murine GFP-IRSp53 were seeded as single cells on Matrigel and fixed after 12 hours. Cells were processed for epifluorescence to visualize GFP-IRSp53 (green) and stained with anti-EPS8 (red) and Dapi (blue). Arrows indicate EPS8 and IRSp53 co-localization at nascent AMIS; arrowheads indicate Eps8 and IRSp53 enrichment in vesicle-like structures. Scale bar, 10 µm.

C) MDCK (upper panels) or MDCK-expressing murine GFP-IRSp53 (lower panels) were seeded as single cells on Matrigel and mature cysts were fixed after 6 days. Cysts were stained with anti-EPS8 (red), FITC-phalloidin to visualize F-actin (green) and Dapi (blue) (upper panels) or processed for epifluorescence to visualize GFP-IRSp53 (green), and stained with anti-EPS8 (red) and Dapi (blue) (lower panels). Arrows indicate EPS8, F-actin and IRSp53 enrichment at the apical/ luminal side of the cysts. Scale bar, 10 µm.

D) Caco-2 (upper panels), Caco-2 infected with GFP (central panels) or Caco-2 infected with GFP-Eps8 were embedded as single cells in Matrigel/Collagen matrix and cysts were fixed after 7 days. Cysts were stained with anti-Eps8 (green), rhodamine-phalloidin to visualize F-actin (red), anti-aPKC (magenta) and Dapi (blue) (upper panels) or processed for epifluorescence to visualize GFP or GFP-EPS8 (green), and stained with rhodamine-phalloidin to visualize F-actin (red), anti-aPKC (magenta) and Dapi (blue) (central and lower panels). Arrows indicate EPS8, F-actin and aPKC enrichment at the apical/luminal side of the cysts. Scale bar, 20 µm.

Supplementary Figure 6

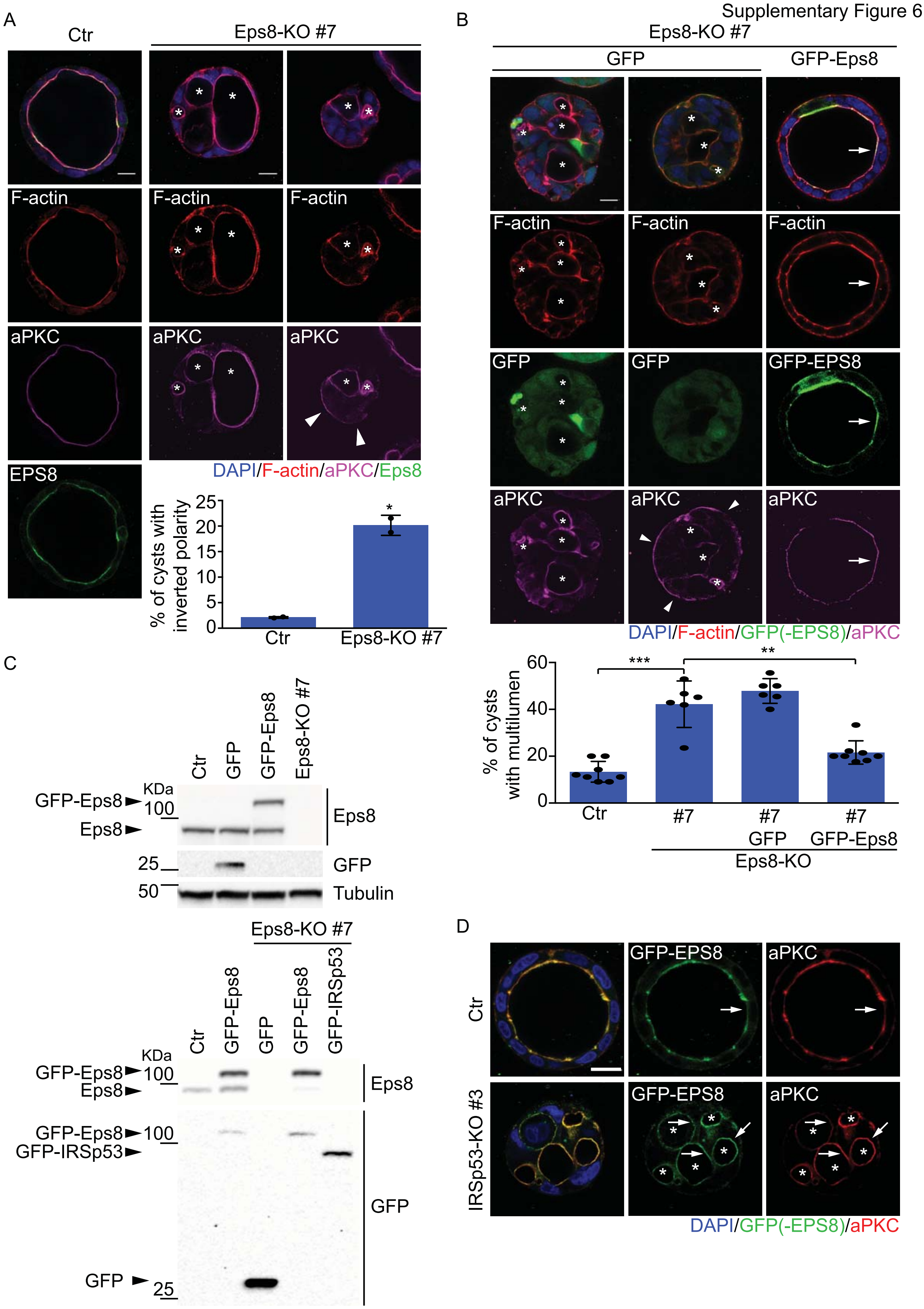

Supplementary Figure 6

The loss of Eps8 leads to the formation of Caco-2 cysts with multiple lumens

A) Caco-2 control (Ctr) or Eps8-KO (clone #7), were embedded as single cells into Matrigel/Collagen matrix and left growing to form cysts. Cysts were fixed and stained with anti-EPS8 (green), rhodamine-phalloidin to detect F-actin (red), anti-a-PKC (magenta), and Dapi (blue). Asterisks and arrowheads indicate multiple lumens and inverted polarity, respectively. Scale bar, 20 µm. Bottom: Quantification of the number of cysts with inverted polarity. Data are expressed as mean ± SD. At least 15 cysts/ experiment were analysed in n=2 independent experiments. P value, Student's t-test two-tailed. *p < 0.05. Source data are provided as a Source Data file. Quantification of multi-lumen cysts is shown in B (bottom graph).

B) Caco-2 Eps8-KO #7, infected with GFP or GFP-Eps8, were embedded as single cells into Matrigel/Collagen matrix and left growing to form cysts. Cysts were fixed, processed for epifluorescence to visualize GFP or GFP-EPS8 (green), and stained with rhodamine-phalloidin to visualize F-actin (red), anti-aPKC (magenta), and Dapi (blue). Asterisks and arrowheads indicate multi-lumens and inverted polarity, respectively. Arrows indicate the enrichment of Eps8, F-actin, and a-PKC at the apical/luminal side of Eps8-KO cysts reconstituted with GFP-EPS8. Scale bar, 20 µm. Bottom: Quantification of the number of cysts with multiple lumens, in comparison to Caco-2 control cells (C2 Ctr) and Caco-2 EPS8-KO #7. Data are expressed as mean ± SD. At least 15 cysts/experiment were analysed in n=2 independent experiments. P value, Student's t-test two-tailed. **p < 0.01; ***p < 0.001. Source data are provided as a Source Data file.

C) The expression levels of GFP, GFP-Eps8, GFP-IRSp53, Eps8, and tubulin were analysed by immunoblotting in Caco-2 control (Ctr) and Caco-2 Eps8-KO #7 to detect Eps8 loss in Caco-2 #7 cells and the expression of the GFP-recombinant proteins.

D) Cysts from Caco-2 control (Ctr) or IRSp53-KO (clone #3) cells infected with GFP-Eps8 were fixed and processed for epifluorescence to visualize GFP-Eps8 or stained with anti-a-PKC (Red), and

Dapi (blue). Arrows indicate GFP-Eps8 and aPKC luminal localization, asterisks indicate multiple lumens. Scale bar, 20 μm.

Supplementary Figure 7

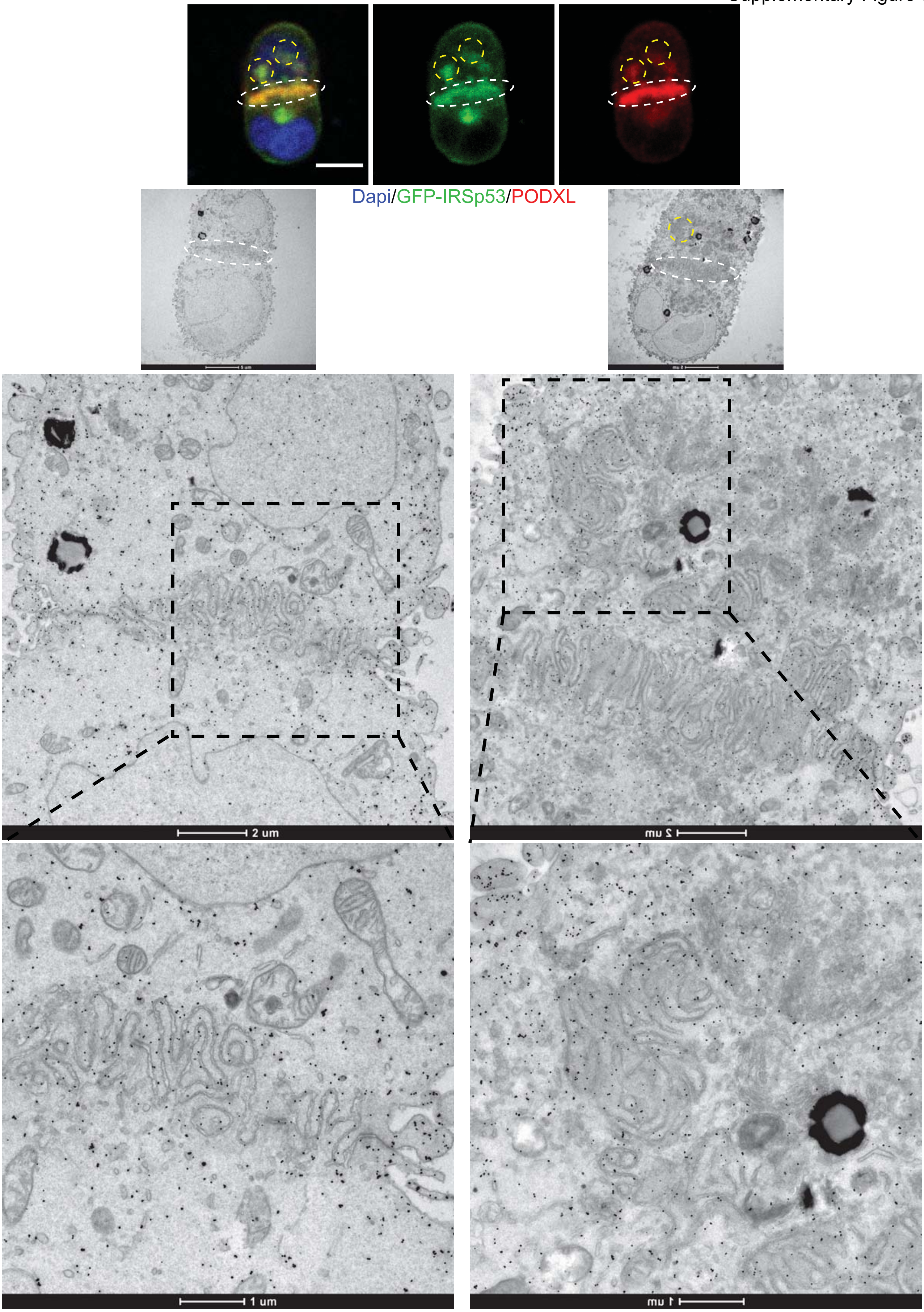

Supplementary Figure 7

Immune-TEM analysis of control and IRSp53-KO MDCK at two-cells stage of cystogenesis

A) MDCK cells expressing GFP-IRSp53 were seeded as single cells on Matrigel-coated gridded coverslips, fixed and processed to visualized GFP-IRSp53, stained with anti-PODXL (red), and Dapi (blue) and analysed by confocal microscope as described in Figure 7B. Two-cell stage cysts were identified on grids by confocal microscopy (upper images, scale bar, 10 µm) and processed for EM analysis with immune-gold labelling against GFP (middle images and lower magnifications). Dashed line white and yellow circles highlight the intervening plasma membranes and vesicle like structures respectively. Scale bar of the EM micrographs are 5 µm, and 2µm and 1 µm in the magnified images.

Supplementary Figure 8

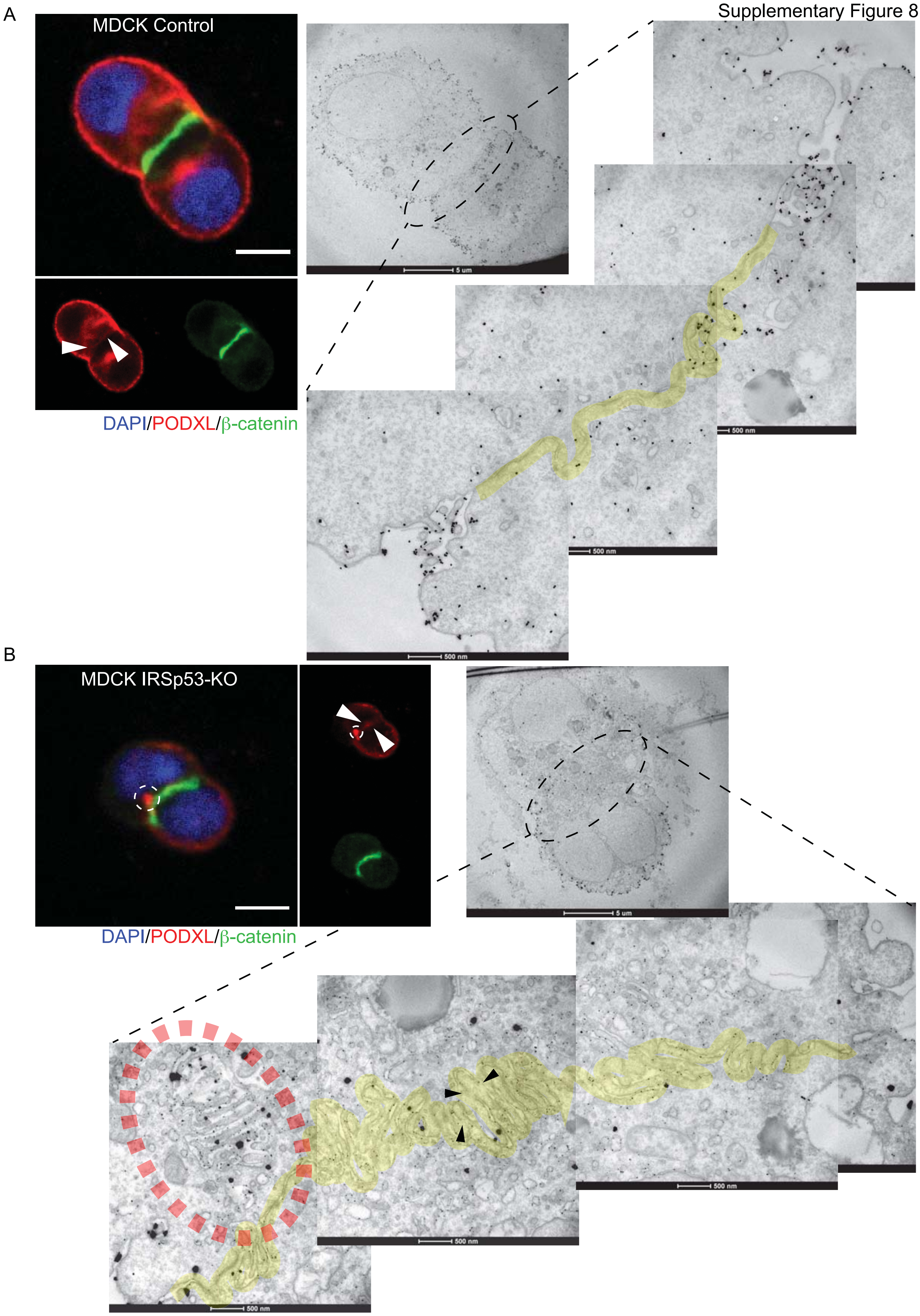

Supplementary Figure 8

TEM analysis of control and IRSp53-KO MDCK at two-cells stage of cystogenesis

A) Control MDCK cells were seeded, stained with anti-ß-catenin (green), anti-PODXL (red), and Dapi (blue) and analysed by confocal microscope as described in Figure 7B. Two-cell stage cysts were identified on grids by confocal microscopy (left image). Arrowheads indicate initial accumulation of PODXL at the nascent AMIS. Scale bar, 10 µm. Cysts were processed for EM analysis with immune-gold labelling against PODXL (middle image). Yellow transparent ribbon highlights the intervening plasma membranes.

B) IRSp53-KO MDCK were treated and analysed exactly as described above. Withe arrowheads (in the IF image) indicate initial accumulation of PODXL at the nascent AMIS. Dashed white (in the IF image) and red (in the EM micrograph) circles highlight the ectopic lumen (or apical vacuole) in IRSp53-KO cells. Arrowheads indicate the inter-cytoplasmic bridges in the PMs. Scale bar of the EM micrographs are 5 µm, and 500 nm in the magnified images.

Supplementary Figure 9

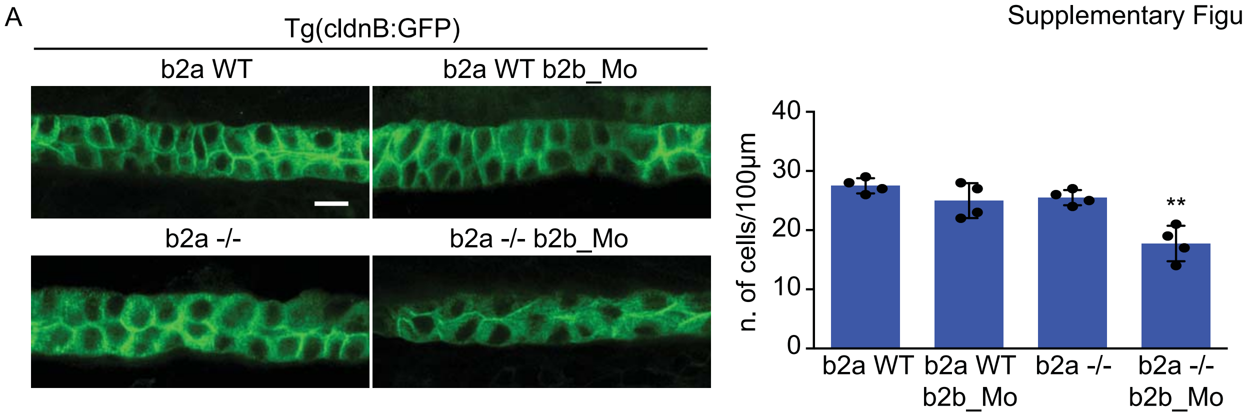

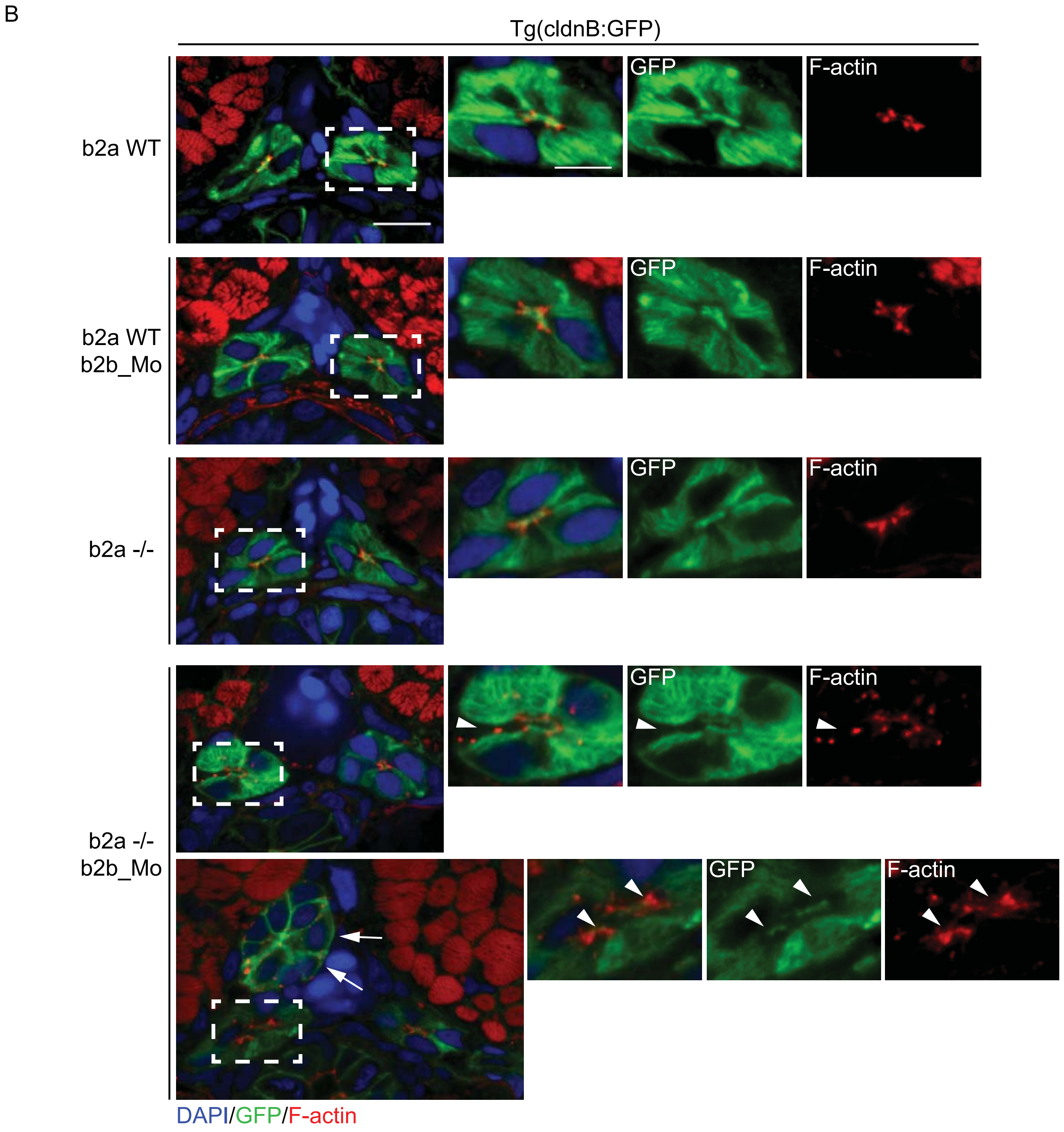

Supplementary Figure 9

Pronephric duct in developing zebrafish after concomitant loss of *baiap2a* and *2b*

A) Digital light sheet confocal images of 72 hpf pronephric ducts. Embryos obtained by either WT treated with scramble oligo (b2a WT), *baiap2a* mutant (b2a -/-), *baiap2b* morphant (b2a WT b2b_Mo) and *baiap2a* mutant *baiap2b* morphant (b2a -/- b2b_Mo) females, in Tg(CldnB:GFP) genetic background, were fixed and mounted in agarose. Samples were stained with anti-GFP (green). Scale bars, 10 µm. Right: The number of nuclei/100µm was counted in different area of the PD in each genetic background. Data are expressed as mean ± SD. At least 4 different area of the PD of n=4 embryos in each genetic background were analysed in n= 1 experiment. P value, Student's t-test two-tailed. **p < 0.01. Source data are provided as a Source Data file.

B) Confocal images of 72 hpf pronephric ducts. Embryos obtained by either WT treated with scramble oligo (b2a WT), *baiap2a* mutant (b2a -/-), *baiap2b* morphant (b2a WT b2b_Mo) and *baiap2a* mutant *baiap2b* morphant (b2a -/- b2b_Mo) females, in Tg(CldnB:GFP) genetic background, were fixed and mounted in agarose. Samples were stained with anti-GFP (green), rhodamine-phalloidin to detect F-actin (red) and Dapi (blue). Right: 2x magnification of the dashed line areas on the left. Arrows and arrowheads indicate the ectopic-duct structure and the luminal aberrations (open lumen, multi-lumen) respectively in b2a -/- b2b_Mo embryo. ~67% of the b2a -/- b2b_Mo embryos show such defects (n = 8/12). Scale bar, 10 µm (5 µm in the right panels).

Supplementary Figure 10

A

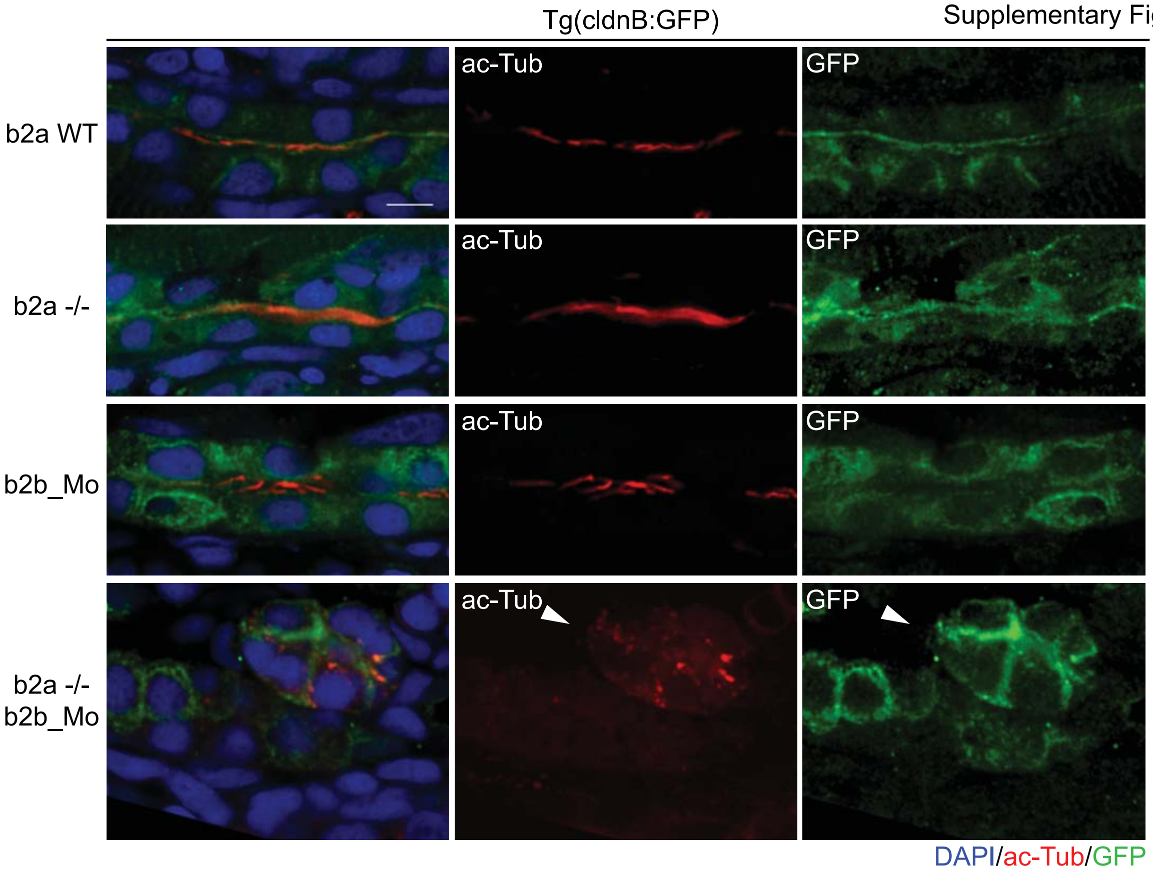

B

ID	Cortical thinning	Architectural tubular disarray	Tubular anisokaryosis*	Vascular lacunae	Glomerular pseudo-aneurysm	Intratubular cast	Total score	Average
WT1 M	0	0	0	1	0	1	2	1,75
WT2 M	0	1	0	1	0	1	3	
WT3 M	0	0	0	2	0	0	2	
WT4 M	0	1	0	1	0	0	2	
WT1 F	0	0	0	0	0	0	0	
WT2 F	0	1	0	0	0	0	1	
WT3 F	1	0	0	1	0	1	3	
WT4 F	0	0	0	0	0	1	1	
KO1 M	0	1	0	0	0	0	1	10
KO2 M	1	2	1	0	0	1	5	
KO3 M	1	2	1	0	0	0	4	
KO4 M	3	3	2	2	2	2	14	
KO5 M	1	2	2	1	2	2	10	
KO6 M	1	2	2	1	2	2	10	
KO1 F	2	3	2	2	2	2	13	
KO2 F	2	3	2	2	3	2	14	
KO3 F	3	3	3	3	2	3	17	
KO4 F	1	2	2	2	2	2	11	
KO5 F	2	3	2	2	0	2	11	
KO6 F	3	3	2	0	1	1	10	
KO7 F	0	2	1	0	0	2	5	
KO8 F	2	3	3	2	2	2	14	
KO9 F	1	3	2	3	2	3	14	
KO10 F	1	2	2	0	1	1	7	

0 = absent; 1 = focal presence; 2 = multifocal presence; 3 = diffuse presence
Tubular anisokaryosis*: irregular distribution and nuclear shape in the tubules

Supplementary Figure 10

Structural defects of pronephric duct in zebrafish and of adult kidney in WT and IRSp53-KO mice

A) Confocal analysis of 72 hpf pronephric ducts. Embryos obtained by either WT treated with scramble oligo (b2a WT), *baiap2a* mutant (b2a -/-), *baiap2b* morphant (b2a WT b2b_Mo) and *baiap2a* mutant *baiap2b* morphant (b2a -/- b2b_Mo) females, in Tg(CldnB:GFP) genetic background, were fixed, processed and paraffin embedded. Slices were stained with anti-GFP (green), anti-acetylated-Tubulin (ac-Tub, red) and Dapi (blue). Arrowhead indicates the irregular distribution of acetylated-tubulin in the extra-duct structure of b2a -/- b2b_Mo embryo pronephric duct. Scale bar, 10 μm. B) Detailed analysis of renal defects. Kidneys from adult (4 and 8 months) mice were analysed for the defect described in the table and a score between 0 (absent) and 3 (diffuse presence) was assigned. Total scores represent the sum of the scoring for each of the described defect. The average represents the Renal Damage Score (see also Figure 8B). WT (n = 8; 4M, 4F) and IRSp53-KO (n = 16; 6M, 10F).

Supplementary Figure 11

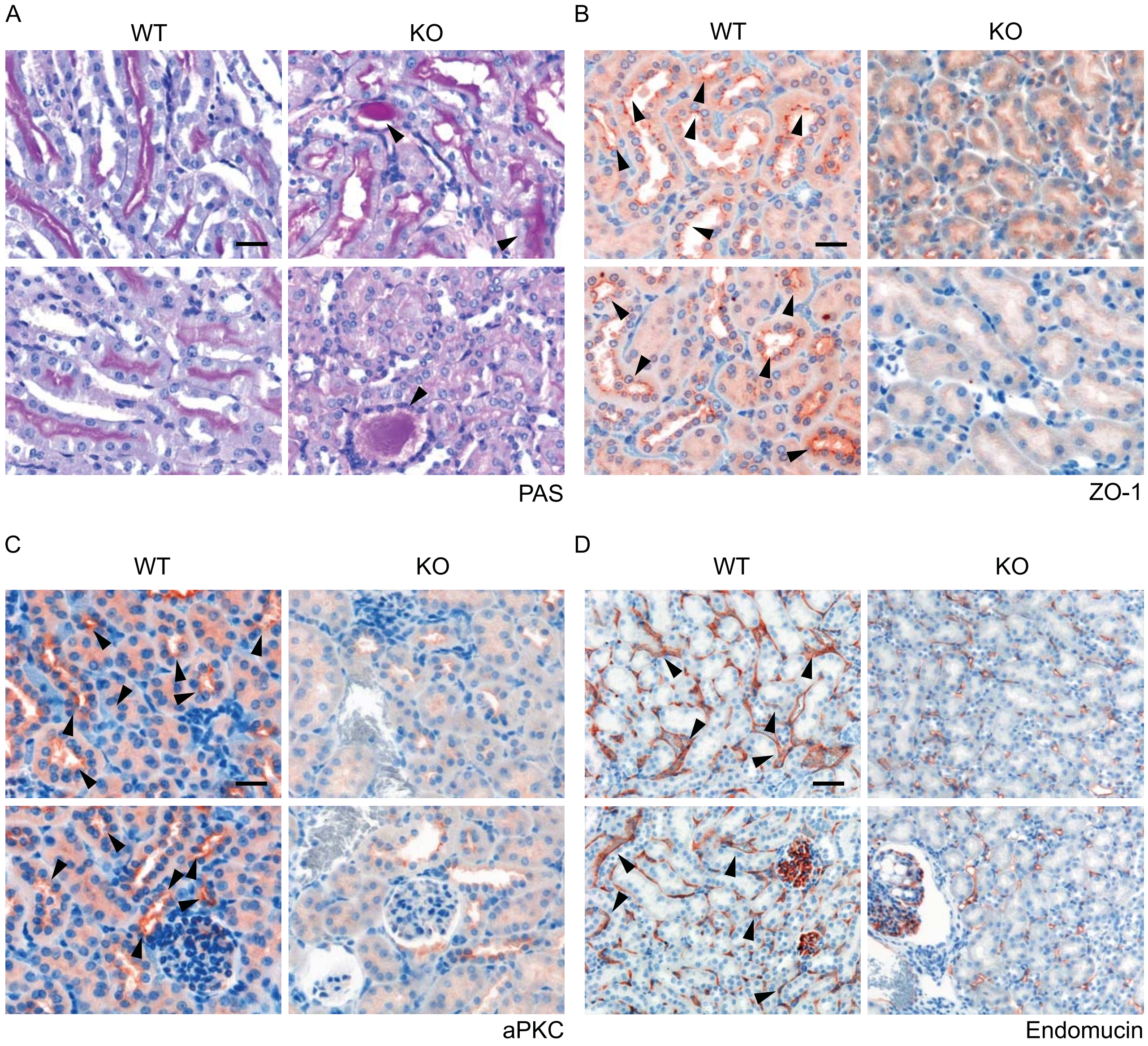

Supplementary Figure 11

Genetic removal of IRSp53 leads to polarity defects in mouse kidney.

A) Periodic acid-Schiff (PAS) analysis in kidney from IRSp53 WT and KO mice. Arrowheads indicate the luminal accumulation of eosinophilic acellular material. Scale bar, 50 µm. B) IHC analysis of ZO-1 expression and localization in kidney from IRSp53 WT and KO mice. Arrowheads indicate ZO-1 apical/luminal enrichment in WT kidney tubules. Scale bar, 50 µm. C) IHC analysis of aPKC expression and localization in kidney from IRSp53 WT and KO mice. Arrowheads indicate aPKC apical/luminal enrichment in WT kidney tubules. Scale bar, 50 µm. D) IHC analysis of endomucin expression and localization in kidney from IRSp53 WT and KO mice. Arrowheads indicate endomucin basal enrichment in WT kidney tubules. Scale bar, 50 µm.

References

1 Vega-Salas, D. E., Salas, P. J., Gundersen, D. & Rodriguez-Boulan, E. Formation of the apical pole of epithelial (Madin-Darby canine kidney) cells: polarity of an apical protein is independent of tight junctions while segregation of a basolateral marker requires cell-cell interactions. *J Cell Biol* **104**, 905-916, doi:10.1083/jcb.104.4.905 (1987).

2 Vega-Salas, D. E., Salas, P. J. & Rodriguez-Boulan, E. Exocytosis of vacuolar apical compartment (VAC): a cell-cell contact controlled mechanism for the establishment of the apical plasma membrane domain in epithelial cells. *J Cell Biol* **107**, 1717-1728 (1988).
