# Supplementary material for: IRSp53 controls plasma membrane shape and polarized transport at the nascent lumen in epithelial tubules
Source: Nat Commun. 2020 Jul 14;11:3516. doi: 10.1038/s41467-020-17091-x (PMC7360740; doi:10.1038/s41467-020-17091-x)
Supplement: Supplementary file 3 — Description of Additional Supplementary Files [file 41467_2020_17091_MOESM3_ESM.pdf]

Description of Additional Supplementary Files

File Name: Supplementary Movie 1
Description: **IRSp53 precedes PODXL localization at the apical membrane since the first cytokinesis of 3D cyst development.** A) Time lapse of GFP-IRSp53 and RFP-PODXL during early phases of cystogenesis. MDCK expressing GFP-IRSp53 and RFP-PODXL were seeded as single cells on a Matrigel. 6 hours after seeding cells were subjected to time-lapse analysis using Confocal Spinning Disk microscope. Images (Bright Field, GFP and RFP channel respectively) were acquired every 5 minutes for 15 hours. White arrowheads indicate GFP-IRSp53 accumulation at cleavage furrow immediately after the first cell division; yellow arrowheads indicate enrichment of RFP-PODXL at nascent AMIS at later stages. Scale bar, 10 µm.

File Name: Supplementary Movies 2-3
Description: **Trafficking of IRSp53 positive vesicular-like structure emerging from peripheral plasma membrane.** MDCK expressing GFP-IRSp53 were seeded as single cells on a Matrigel. 6 hours after seeding cells were subjected to time-lapse analysis using Confocal Spinning Disk microscope. Images (Bright Field and GFP channel respectively) were acquired every 5 minutes for 15 hours. White circles indicate GFP-IRSp53 vesicular-like structures that emerge from the peripheral plasma membrane and move toward the inner (Movie S2) or the forming apical side at cleavage furrow immediately after the first cell division (Movie S3). Scale bar, 10 µm.

File Name: Supplementary Movie 4
Description: **Loss of IRSp53 alters facing PMs at the nascent AMIS.** Electron Microscopy Tomography of MDCK Ctr (left) and IRSp53-KO (right) two-cells stage intervening membranes during early cystogenesis. Cells were seeded and processed as in Figure 7B. Dashed red and black lines define the plasma membrane, while the arrowhead indicates the inter-cytoplasmic bridge of the intervening membranes during AMIS formation at 2-cells stage.

File Name: Supplementary Movie 5

Description: **Genetic removal of IRSp53 leads to defects in *zebrafish* kidney development.** 3D rendering of deconvoluted confocal images of pronephric ducts of Tg(CldnB:GFP)-WT, treated with scramble oligo (b2a WT), or Tg(CldnB:GFP)-*baiap2a* mutant zebrafish strain treated with splice-blocking and translational-blocking morpholinos (b2a -/- b2b_Mo).
