# Supplementary material for: IRSp53 controls plasma membrane shape and polarized transport at the nascent lumen in epithelial tubules
Source: Nat Commun. 2020 Jul 14;11:3516. doi: 10.1038/s41467-020-17091-x (PMC7360740; doi:10.1038/s41467-020-17091-x)
Supplement: Supplementary file 9 — Reporting Summary [file 41467_2020_17091_MOESM9_ESM.pdf]

nature research

Corresponding author(s): Giorgio Scita

Last updated by author(s): 2020 05 14

Reporting Summary

Nature Research wishes to improve the reproducibility of the work that we publish. This form provides structure for consistency and transparency in reporting. For further information on Nature Research policies, see our Editorial Policies and the Editorial Policy Checklist.

Statistics

For all statistical analyses, confirm that the following items are present in the figure legend, table legend, main text, or Methods section.

n/a	Confirmed	
☐	☒	The exact sample size (*n*) for each experimental group/condition, given as a discrete number and unit of measurement
☐	☒	A statement on whether measurements were taken from distinct samples or whether the same sample was measured repeatedly
☐	☒	The statistical test(s) used AND whether they are one- or two-sided<br>*Only common tests should be described solely by name; describe more complex techniques in the Methods section.*
☒	☐	A description of all covariates tested
☐	☒	A description of any assumptions or corrections, such as tests of normality and adjustment for multiple comparisons
☐	☒	A full description of the statistical parameters including central tendency (e.g. means) or other basic estimates (e.g. regression coefficient) AND variation (e.g. standard deviation) or associated estimates of uncertainty (e.g. confidence intervals)
☒	☐	For null hypothesis testing, the test statistic (e.g. *F*, *t*, *r*) with confidence intervals, effect sizes, degrees of freedom and *P* value noted<br>*Give P values as exact values whenever suitable.*
☒	☐	For Bayesian analysis, information on the choice of priors and Markov chain Monte Carlo settings
☒	☐	For hierarchical and complex designs, identification of the appropriate level for tests and full reporting of outcomes
☒	☐	Estimates of effect sizes (e.g. Cohen's *d*, Pearson's *r*), indicating how they were calculated

Our web collection on statistics for biologists contains articles on many of the points above.

Software and code

Policy information about availability of computer code

Data collection	- IHC images were collected with:<br>1) Zeiss Axio Scope A1 optical microscope (ZEN 2 Blue Edition v2.0.0.0, Zeiss).<br>2) Olympus BX63 Upright microscope (MetaMorph RGB v7.8.10.0, Molecular Devices, LLC)<br>3) Aperio ScanScope (v12.2.2, Leica Biosystems).<br>See Methods for details.<br>- Immuno-Blotting acquisitions were performed with Image Lab software (v3.0, Bio-Rad Laboratories).<br>- Immunofluorescence data was collected with Upright Olympus BX51 FL (MetaVue v7.5.0.0, MDS Analytical Technologies)<br>- Confocal acquisitions were performed with Leica TCS SP2 AOBS (LCS v2.61, Leica Biosystems) , TCS SP5 (LAS AF v2.7.3, Leica Biosystems), TCS SP8 (LASX v3.5.2.18963 or v3.5.1.18803, Leica Biosystems), UltraVIEW VoX (Volocity software Improvision v6.3.1, Perkin Elmer) spinning disk confocal unit or Confocal Spinning Disk microscope (Olympus CellSens Dimension 1.18 software-Build 16686, Olympus).<br>See Methods for details.<br>- RT-qPCR data were collected with 7500 Software (v2.0.6, Applied Byosistems).
Data analysis	- IHC images were analysed with Aperio ScanScope (v12.2.2, Leica Biosystems) and/or ImageJ (v1.52, NIH).<br>- Immuno Blotting analysis was performed with Image Lab software (v3.0, Bio-Rad Laboratories) and/or Image J (v1.52, NIH).<br>- Immunofluorescence (widefield and confocal) analysis was performed with Image J (v1.52, NIH).<br>- Confocal images deconvolution was performed with a Huygens Essential software (v19.10, SVI)<br>- Mass Spectrometry raw data were processed with MaxQuant (v1.4.1.2.) and quantitative analysis was performed using Perseus (v1.5.1.6).

- Statistical analysis of RT-qPCR experiments, IB and IF quantifications were performed with Excel Office 2019 (v16.16.21, Microsoft) or with Prism (v6.0c, Graph Pad).
See Methods for details.

For manuscripts utilizing custom algorithms or software that are central to the research but not yet described in published literature, software must be made available to editors and reviewers. We strongly encourage code deposition in a community repository (e.g. GitHub). See the Nature Research guidelines for submitting code & software for further information.

Data

Policy information about availability of data

All manuscripts must include a data availability statement. This statement should provide the following information, where applicable:

- Accession codes, unique identifiers, or web links for publicly available datasets
- A list of figures that have associated raw data
- A description of any restrictions on data availability

All full scan immunoblots, gels and all data set used to generate each of the graph presented in the work are provided in the Source Data file. The authors declare that all of the data that support the findings of this study are available within the paper and its Supplementary Information files, and from the corresponding authors upon reasonable request.
Proteomic data have been deposited and are available at the PeptideAtlas repository (http://www.peptideatlas.org/PASS/PASS01464).

Field-specific reporting

Please select the one below that is the best fit for your research. If you are not sure, read the appropriate sections before making your selection.

[x] Life sciences [ ] Behavioural & social sciences [ ] Ecological, evolutionary & environmental sciences

For a reference copy of the document with all sections, see nature.com/documents/nr-reporting-summary-flat.pdf

Life sciences study design

All studies must disclose on these points even when the disclosure is negative.

Sample size	Sample size was determined according to previously conducted experiments and determined to be adequate based on the magnitude and consistency of measurable differences between groups. The number of experiments (n) is indicated in each figure legend.
Data exclusions	No data was excluded.
Replication	Each of the finding has been reproduced at least 3 times unless otherwise specified
Randomization	No randomization was applied
Blinding	Investigators were blinded to group allocation during analysis of multi-lumen vs single lumen or inverted polarity determination in 3D cysts, localization of RFP-RAB35 in Ctr vs IRSp53-KO 3D cysts, podocalyxin trafficking assays, determination of cytoplasmic bridges from EM images, analysis of kidney samples form WT vs IRSp53-KO mice or Ctr vs Baiap2a/b mutants zebrafish .<br>For the other experiments, the investigators were not blinded to experimental groups during data collection and analysis. Data reported in this latter case is not subjective.

Reporting for specific materials, systems and methods

We require information from authors about some types of materials, experimental systems and methods used in many studies. Here, indicate whether each material, system or method listed is relevant to your study. If you are not sure if a list item applies to your research, read the appropriate section before selecting a response.

Materials & experimental systems

n/a	Involved in the study	
[ ]	[x]	Antibodies
[ ]	[x]	Eukaryotic cell lines
[x]	[ ]	Palaeontology and archaeology
[ ]	[x]	Animals and other organisms
[ ]	[x]	Human research participants
[x]	[ ]	Clinical data
[x]	[ ]	Dual use research of concern

Methods

n/a	Involved in the study	
[x]	[ ]	ChIP-seq
[x]	[ ]	Flow cytometry
[x]	[ ]	MRI-based neuroimaging

Antibodies

Antibodies used

Primary antibodies
1) homemade (IFOM) mouse monoclonal anti-IRSp53 (epitope: Full Length protein aa 1-521, Homo sapiens S Isoform) - WB 1:150; IF 1:150; IHC 1:150/1:200
2) anti-IRSp53 rabbit SIGMA, HPA023310 - IHC 1:50
3) anti-IRSp53 rabbit from Hans-Jürgen Kreienkamp, University of Hamburg - WB 1:200; IF 1:100; IHC 1:30
4) anti-vinculin mouse SIGMA, V9131 - WB 1:1000
5) anti-GFP rabbit SIGMA, G1544 - WB 1:500
6) anti-GFP mouse Abcam, ab6556 - Immuno-EM
7) anti-GFP chicken Abcam, ab13970 - IF 1:1000
8) anti-GP135/podocalyxin mouse DSHB, Iowa University, 3F2/D8 - WB 1:500; IF 1:400; Immuno-EM 1:100
9) anti-Eps8 mouse BD-Transduction Lab, 610144 - WB 1:1000; IF 1:1000 (Caco2 cysts 1:100)
10) anti-RAB7 rabbit Cell Signaling, 2094 - IF 1:150
11) anti-ZO1 rabbit Invitrogen, 402200 - IF 1:400 (Caco2 cysts 1:100)
12) anti-mouse ZO-1 rabbit Genetex GTX108592 - IHC 1:100
13) anti-PKC ζ rabbit SCBT sc-216 - IF (Caco2 cyts) 1:100; IHC 1:100
14) anti-ß-catenin rabbit SIGMA, PLA2030 - IF 1:500 (Caco2 cysts 1:100); Immuno-EM 1:100
15) homemade (IFOM) mouse anti-Myc 9E10 - WB 1:1000; IF 1:1000
16) anti-EEA1 goat SCBT, sc-6415 - IF 1:150
17) anti-LAMP1 rabbit SIGMA, L1418 - IF 1:100
18) anti-Giantin rabbit BioLegend, 924302 - IF 1:100
19) anti-Flag mouseSIGMA, F3165 - WB 1:1000; IF 1:1000; IP 5µg/mg
20) anti-Rab35 rabbit Cell Signaling, 9690 - WB 1:500
21) anti-tubulin mouse SIGMA, T5168 - WB 1:1000; IF 1:1000
22) anti-acetyl-α-tubulin mouse Abcam, Ab24610 - IF 1:500
23) anti-Endomucin rat Abcam, ab106100 - IHC 1:100

Secondary antibodies
1) anti-rabbit IgG, HRP-linked antibody Cell Signaling, 7074 - WB 1:2500
2) anti-mouse IgG, HRP-linked antibody Cell Signaling, 7076 - WB 1:2500
3) anti-rabbit Fab' fragments coupled to 1.4nm gold particles goat Nanoprobes, #2004 - Immuno-EM 1:100
4) anti-mouse Fab' fragments coupled to 1.4nm gold particles goat Nanoprobes, #2002- Immuno-EM 1:100
5) Alexa Fluor 488 Donkey anti rabbit ThermoFisher, A-21206 - IF 1:400 (Caco2 cysts 1:100)
6) Alexa Fluor 488 Donkey anti mouse ThermoFisher, A-21202 - IF 1:400 (Caco2 cysts 1:100)
7) Alexa Fluor 488 Goat anti chicken Thermofisher, A-11039 - IF 1:400
8) Alexa Fluor 647 Donkey anti rabbit ThermoFisher, A-31573 - IF 1:400 (Caco2 cysts 1:100)
9) Alexa Fluor 647 Donkey anti mouse ThermoFisher, A-31571 - IF 1:400 (Caco2 cysts 1:100)
10) Cy3 Donkey anti rabbit Jackson Immunoresearch, 711-165-152 - IF 1:400 (Caco2 cysts 1:100)
11) Cy3 Donkey anti mouse Jackson Immunoresearch, 715-165-150 - IF 1:400 (Caco2 cysts 1:100)
12) Cy3 Donkey anti goat Jackson Immunoresearch, 705-165-147 - IF 1:400
13) Phalloidin TRITC SIGMA, P1951- IF 1:50 (Caco2 cysts 1:10)
14) Phalloidin FITC SIGMA, P5282- IF 1:50 (Caco2 cysts 1:10)

Validation

Primary antibodies
1) IRSp53 antibody was already described and validated (Disanza A. et al, EMBO, 2013)
2) https://www.sigmaaldrich.com/catalog/product/sigma/hpa023310?lang=it&region=IT
3) Validated in house expressing recombinant tagged ZF Baiap2a and Baiap2b in IRSp53 KO MEFs (IF and WB) or comparing b2a WT vs b2a-/- b2b_Mo Zebrafish embrios (IHC)
4) https://www.sigmaaldrich.com/catalog/product/sigma/v9131?lang=it&region=IT
5) https://www.sigmaaldrich.com/catalog/product/sigma/g1544?lang=it&region=IT
6) https://www.abcam.com/gfp-antibody-ab6556.html
7) https://www.abcam.com/gfp-antibody-ab13970.html
8) https://dshb.biology.uiowa.edu/3F2-D8_2
9) https://www.bdbiosciences.com/us/reagents/research/antibodies-buffers/cell-biology-reagents/cell-biology-antibodies/purified-mouse-anti-eps8-15eps8/p/610144
10) https://www.cellsignal.com/products/primary-antibodies/rab7-antibody/2094
11) https://www.thermofisher.com/antibody/product/ZO-1-Antibody-Polyclonal/40-2200
12) https://www.genetex.com/Product/Detail/ZO-1-antibody/GTX108592
13) https://www.scbt.com/it/p/pkc-zeta-antibody-c-20
14) https://www.sigmaaldrich.com/catalog/product/sigma/pla0230?lang=it&region=IT
15) Validated in house expressing recombinant tagged construts (IF and WB)
16) https://www.scbt.com/it/p/eea1-antibody-n-19
17) https://www.sigmaaldrich.com/catalog/product/sigma/l1418?lang=it&region=IT
18) https://www.biolegend.com/en-us/products/anti-giantin-antibody-11064
19) https://www.sigmaaldrich.com/catalog/product/sigma/f3165?lang=it&region=IT

20) https://www.cellsignal.com/products/primary-antibodies/rab35-antibody/9690
21) https://www.sigmaaldrich.com/catalog/product/sigma/t5168?lang=it&region=IT
22) https://www.abcam.com/alpha-tubulin-acetyl-k40-antibody-6-11b-1-ab24610.html
23) https://www.abcam.com/endomucin-antibody-v7c71-ab106100.html

Secondary antibodies
1) https://www.cellsignal.com/products/secondary-antibodies/anti-rabbit-igg-hrp-linked-antibody/7074
2) https://www.cellsignal.com/products/secondary-antibodies/anti-mouse-igg-hrp-linked-antibody/7076
3) https://www.nanoprobes.com/products/Nanogold-Antibody-Conjugates.html
4) https://www.nanoprobes.com/products/Nanogold-Antibody-Conjugates.html
5) https://www.thermofisher.com/antibody/product/Donkey-anti-Rabbit-IgG-H-L-Highly-Cross-Adsorbed-Secondary-Antibody-Polyclonal/A-21206
6) https://www.thermofisher.com/antibody/product/Donkey-anti-Mouse-IgG-H-L-Highly-Cross-Adsorbed-Secondary-Antibody-Polyclonal/A-21202
7) https://www.thermofisher.com/antibody/product/Goat-anti-Chicken-IgY-H-L-Secondary-Antibody-Polyclonal/A-11039
8) https://www.thermofisher.com/antibody/product/Donkey-anti-Rabbit-IgG-H-L-Highly-Cross-Adsorbed-Secondary-Antibody-Polyclonal/A-31573
9)https://www.thermofisher.com/antibody/product/Donkey-anti-Mouse-IgG-H-L-Highly-Cross-Adsorbed-Secondary-Antibody-Polyclonal/A-31571
10) https://www.jacksonimmuno.com/catalog/products/711-165-152
11) https://www.jacksonimmuno.com/catalog/products/715-165-150
12) https://www.jacksonimmuno.com/catalog/products/705-165-147
13) https://www.sigmaaldrich.com/catalog/search?term=P1951&interface=All&N=0&mode=partialmax&lang=it&region=IT&focus=product
14) https://www.sigmaaldrich.com/catalog/search?term=P5282&interface=All&N=0&mode=partialmax&lang=it&region=global&focus=product

Eukaryotic cell lines

Policy information about cell lines

Cell line source(s)	MDCK, Caco-2 and Hela cells were from the American Type Culture Collection (ATCC); 293T cells were from Interlab Cell Line Collection (ICLC); MDCK IRSp53 shRNAmir inducible cells were generated in the lab of Dr. Ann Musch (Albert Einstein College of Medicine, NY, USA).
Authentication	All cell lines were authenticated at each batch freezing by fingerprinting by IFOM cell culture facility
Mycoplasma contamination	All cell lines are routinely tested for mycoplasma by IFOM cell culture facility, with negative results.
Commonly misidentified lines (See ICLAC register)	No commonly misidentified cell lines were used in the study.

Animals and other organisms

Policy information about studies involving animals; ARRIVE guidelines recommended for reporting animal research

Laboratory animals	Female and male mice between 8 and 32 weeks old were used for all experiments. Mouse lines used: C57Bl6/J and IRSp53 KO in C57Bl6/J background.<br>Zebrafish strains used in this study are AB (referred as wild type), sa11319 obtained from European Zebrafish Resource Center (EZIRC) (referred as baiap2aC201*) and Tg(cldnB:GFP). Female and male embryos between 2h and 96hpf or female and male adults fish between 8 and 78 weeks were used for all the experiments.
Wild animals	This study did not involve wild animals.
Field-collected samples	This study did not involve animals collected from the field.
Ethics oversight	All mouse and zebrafish experiments were conducted in a certified animal facility under the control of the institutional organism for animal welfare and ethical approach to animals in experimental procedures (Cogentech OPBA). All animal studies were conducted with the approval of Italian Minister of Health (598/2015-PR and 219/2016-PR) and were performed in accordance with the Italian law (D.lgs. 26/2014), which enforces Dir. 2010/63/EU (Directive 2010/63/EU of the European Parliament and of the Council of 22 September 2010 on the protection of animals used for scientific purposes).

Note that full information on the approval of the study protocol must also be provided in the manuscript.

Human research participants

Policy information about studies involving human research participants

Population characteristics	Human archival tissue samples for IHC analyses were retrieved from the archives of the Tumor Immunology Laboratory of the University of Palermo, under the Institutional Review Board Approval number 09/2018.
Recruitment	All the samples were collected and handled according to the Helsinki Declaration.
Ethics oversight	Tumor Immunology Laboratory of the University of Palermo, under the Institutional Review Board Approval number 09/2018.

Note that full information on the approval of the study protocol must also be provided in the manuscript.
